# Ivacaftor Reduces Inflammatory Mediators in Upper Airway Lining Fluid From Cystic Fibrosis Patients With a G551D Mutation: Serial Non-Invasive Home-Based Collection of Upper Airway Lining Fluid

**DOI:** 10.3389/fimmu.2021.642180

**Published:** 2021-05-05

**Authors:** Jochen G. Mainz, Christin Arnold, Kara Wittstock, Uta-Christina Hipler, Thomas Lehmann, Carlos Zagoya, Franziska Duckstein, Helmut Ellemunter, Julia Hentschel

**Affiliations:** ^1^ Cystic Fibrosis Center, Brandenburg Medical School (MHB) University, Klinikum Westbrandenburg, Brandenburg an der Havel, Germany; ^2^ CF-Center, Jena University Hospital, Jena, Germany; ^3^ Faculty of Health Sciences, Joint Faculty of the Brandenburg University of Technology Cottbus –Senftenberg, The Brandenburg Medical School Theodor Fontane and the University of Potsdam, Cottbus, Brandenburg an der Havel and Potsdam, Germany; ^4^ Jena University Hospital, Department of Dermatology, Jena, Germany; ^5^ Jena University Hospital, Centre for Clinical Studies (Biometrics), Jena, Germany; ^6^ Medical University of Innsbruck, CF-Center, Innsbruck, Austria; ^7^ Institute of Human Genetics, Leipzig University Hospital, Leipzig, Germany

**Keywords:** cystic fibrosis, CFTR, modulation, inflammation, sampling, cytokines, nasal lavage

## Abstract

In cystic fibrosis (CF) therapy, the recent approval of CF-transmembrane conductance regulator (CFTR) channel modulators is considered to be the major breakthrough. However, the current first-line approach based mainly on pulmonary function to measure effects of the novel therapy, tested by forced expiratory volumes in one second (FEV_1_), provides restricted sensitivity to detect early structural damages. Accordingly, there is a need for new sensitive surrogate parameters. Most interestingly, these should quantify inflammation that precedes a decline of pulmonary function. We present a novel method assessing inflammatory markers in the upper airways’ epithelial lining fluid (ELF) obtained by nasal lavage (NL). In contrast to broncho-alveolar lavage, ELF sampling by NL is an attractive method due to its limited invasiveness which allows repeated analyses, even performed in a home-based setting. In a longitudinal cohort study (ClinicalTrials.gov, Identifier: NCT02311140), we assessed changes of inflammatory mediators in 259 serially obtained nasal lavages taken up to every second day before and during therapy with ivacaftor from ten CF patients carrying a G551D mutation. Patients were trained to sample NL-fluid at home, to immediately freeze and transfer chilled secretions to centers. Neutrophil Elastase, Interleukins IL-1β, IL-6 and IL-8 in NL were quantified. During 8-12 weeks of ivacaftor-treatment, median values of IL-1β and IL-6 significantly declined 2.29-fold (2.97→1.30 pg/mL), and 1.13-fold (6.48→5.72 pg/mL), respectively. In parallel, sweat tests and pulmonary function improved considerably. This is the first study assessing changes of airway inflammation on a day-to-day basis in CF patients receiving a newly administered CFTR-modulator therapy. It proves a decline of airway inflammation during ivacaftor-therapy.

## Introduction

Cystic fibrosis (CF) is the most frequent lethal inherited disease in the Caucasian population and is caused by a defective cystic fibrosis transmembrane conductance regulator gene (*CFTR*). This gene encodes an ion channel located at apical membranes of exocrine glands. Loss or altered function of *CFTR* results in impaired transport of chloride and sodium, bicarbonate, and water. As a consequence, progressive pulmonary destruction by viscous secretions, impaired mucociliary clearance, and pathogen colonization results in a vicious circle of chronic inflammation and leads to premature death in almost 90% of CF patients.

Modulation of the impaired transmembrane conductance regulator (CFTR) protein (1, 2) was a major breakthrough. Initially and for long the most effective modulator treatment, the CFTR potentiator ivacaftor was approved for CF patients carrying the rare G551D *CFTR* mutation (1). It increases Cl^-^ secretion in cultured human CF bronchial epithelia carrying the gating mutation G551D by approximately 10-fold, reaching almost 50% of the secretory potential found in non-CF human bronchial epithelia. Furthermore, ivacaftor reduces excessive Na^+^ and fluid absorption, preventing dehydration of the apical surface and increasing cilia beating in epithelial cultures (3). In CF patients carrying a G551D mutation, ivacaftor improves lung function, pulmonary exacerbation rate, respiratory symptoms, and weight gain when compared to placebo (1, 4). Following this success in rare mutations like G551D (1, 5), research focused on developing modulators for more frequent CFTR mutations. Recently, the triple combination of elexacaftor-tezacaftor-ivacaftor has been approved in the US and some European countries for patients carrying the frequent *CFTR*-mutation F508del (2, 6).

CFTR modulators’ effects on the abnormal and exaggerated inflammation in CF are presently poorly understood. In both, cell and *in vivo* studies, no consensus has been reached as to whether CFTR modulation can control such an abnormal inflammatory response (7–9). Only two studies in humans assessed the effects of ivacaftor on inflammatory marker’s levels in sputum from CF patients carrying the mutation G551D (10, 11), and a more recent one focused on the effect of CFTR-modulator combinations, ivacaftor/lumacaftor and ivacaftor/tezacaftor, on the inflammatory response in serum from F508del-homozygous CF patients (12).

Conclusions from the two studies on ivacaftor effects on the cytokines IL-1β and IL-8 contradict each other, albeit their agreement on the reduced rate of infections by *P.aeruginosa* during the first year of ivacaftor therapy. While Rowe et al. found no significant changes in measures of both inflammation markers at baseline and six months after ivacaftor therapy initiation (11), the longitudinal study by Hisert et al. revealed a decreasing trend in both parameters over a 600-day period (10). In the study involving F508del-homozygous CF patients, only the combination ivacaftor/tezacaftor was observed to reduce IL-1β levels after three months. Interestingly, both combinations, ivacaftor/lumacaftor and ivacaftor/tezacaftor, reduced IL-18 levels in this group of CF patients after the three-month period (12).

Motivated by these discrepancies, in the present study we analyzed early changes in inflammatory markers in shorter intervals. For matters of non-invasiveness and repeatability, we opted for serial home-based nasal lavage (NL) as a means to assess early changes in the upper airway (UAW) fluid of CF patients with a G551D mutation prior to and during novel treatment with ivacaftor. NL has proved to be an easy, non-invasive, and repeatable means to sample NL fluid (NLF) (13, 14), facilitating close monitoring of inflammatory dynamics and assessment of early changes. In a previous study, we used NL to show that concentrations of soluble inflammatory markers result to be higher in CF patients than in healthy controls (15). Furthermore, by means of NL, we have shown that interleukins (IL)-1β, IL-6 and IL-8 are elevated in UAWs colonized by *S.aureus* and *P.aeruginosa* (16) which significantly decreased during antibiotic treatment (17). The primary outcome parameter in most studies for approval of CFTR modulators was FEV_1_. In adult patients with a G551D mutation, FEV_1_ improved by 10-12% predicted, based on an overall FEV_1_ of 40-90% predicted as inclusion criterion (1). However, in early stages of the respiratory disease, FEV_1_ is insensitive to detect changes because airway inflammation and infections precede the decline of pulmonary function (18). Secondly, FEV_1_ fails to sufficiently represent irreversible structural damage and bronchiectasis (19). Consequently, new surrogate parameters are required to assess effects of CFTR modulators.

CF patients’ UAW mucosa is an attractive site for monitoring CF disease status as it exhibits the same epithelial CFTR defect as in the lower airway (LAW) (20, 21). Patients’ nasal histology, regularly displaying inflammatory changes and pathologies in sinonasal CT-scans, has therefore always been a hallmark of CF (20). In previous studies, nasal potential difference measurement proved to be sensitive to quantify effectiveness of ivacaftor in patients with G551D but this electrophysiological method is limited for its complexity (22). On the other hand, direct measurement of inflammatory markers in bronchial epithelial lining fluid obtained through broncho-alveolar lavage (BAL) (18) is limited for its invasiveness.

**Figure 1:**
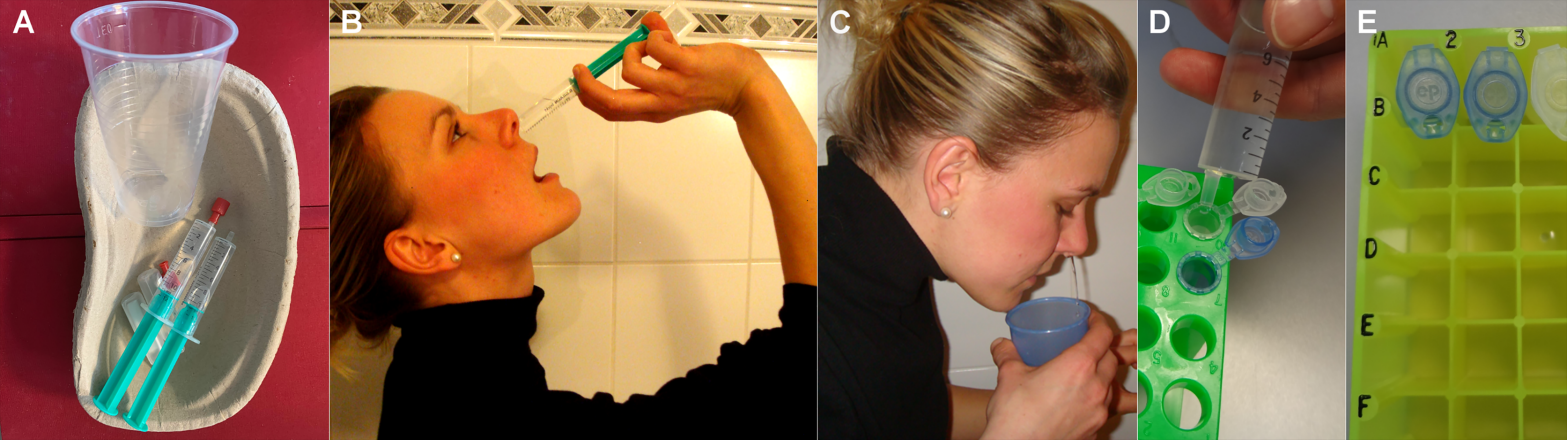
**(A–C)** Method to sample NL-fluid rinsing 10 mL of isotonic saline through each nasal side while closing the soft palate. **(D, E)** home-based aliquotation into disposed reaction tubes, containing 15 µL of protease inhibitor, before freezing.

In a previous cross-sectional study involving 187 CF patients of all ages, we identified NL as the most sensitive sampling technique to monitor UAW-pathogen-colonization (13) compared to nasal swabs. According to our standard operation procedures (SOPs), put forward in a previous publication, NL is performed by instilling 10 mL of isotonic NaCl into each nostril with closed soft palate (**Figures 1A–E**) (14), an easy technique well accepted by almost all of our patients. Consequently, we routinely monitored CF patients’ sinonasal colonization by NL during the last decade at the Jena University CF-center. Hereby, we identified patients manifesting isolated primary sinonasal colonization with *P.aeruginosa* (23). Furthermore, after lung transplantation, we proved pathogen persistence in the UAW, from where they spread to primarily *P.aeruginosa*-free transplanted organs (24). It is noteworthy that we assessed effects of sampling, processing and storage methods on inflammatory markers in NL implementing our findings in SOPs, e.g. a video for researchers and patients (14) facilitating standardization of home-based, day-to-day sampling.

The objective of this study is to examine serial home-based NL as a means to assess early changes in inflammatory markers at shorter intervals in the UAW-fluid of CF patients with a G551D mutation, prior to and during novel treatment with ivacaftor. At the same time, our aim is to shed light on the controversial discussion whether CFTR modulators have anti-inflammatory effects.

## Material and Methods

### Enrollment of Patients

In this longitudinal trial, 10 CF patients with a G551D mutation, newly receiving ivacaftor 150 mg bid, as approved (1), were included in the Jena (Germany) and Innsbruck (Austria) University CF centers. Inclusion criteria were: CF patients’ age ≥ 6 years with a diagnosis confirmed by two positive sweat tests and/or detection of two disease-causing CFTR mutations; one of them being G551D. Patients were required to strictly follow the procedures defined in the SOPs and they and/or their parents gave informed written consent prior to enrolment. Exclusion criteria were: inability to perform NL in a standardized manner as described below, contraindication for ivacaftor, pregnancy or breast-feeding, and participation in another clinical study within 30 days prior to study drug administration.

### Nasal Lavage

Patients were thoroughly trained to perform NL in standardized home-based conditions (**Figures 1A–C**) by using the instruction-video from our publication in Journal of Immunological Methods 2014 (14) and an illustrated SOP sheet. The pooled native NLF was transferred into disposed reaction tubes containing 15 µL of protease inhibitor (**Figures 1D, E** **)** and, within 30 minutes after sampling, stored in a freezer below -18°C until transfer on ice to the centers. Here, the vials were directly stored at - 80°C until performance of laboratory analyses.

Prior to the initiation of ivacaftor therapy, NL was collected at least once a week (mean: 5.5 ± 3.9 samplings/patient) during a mean time of 30.3 ± 19.7 days. During the first month of therapy (days 0-30), NL was collected every second day and once/week from month two onward (mean: 25.9 ± 4.1 samplings/patient). Follow-up range after initiation of ivacaftor: 2.3-16.1 months, (median: 5.7 months).

### Assessment of Inflammatory Mediators

Concentrations of IL-1β, IL-6, and IL-8 were measured in undiluted NL fluid using Milliplex^®^ MAP Kits (Human High Sensitivity HS TCMAG-28K, Merck Millipore; Darmstadt, Germany) and a Bio-Plex^®^ 200 system (Bio-Rad; Hercules, California, USA) according to the manufacturer’s instructions. Measurements of Neutrophil Elastase (NE) were performed using Human PMN Elastase ELISA (DEH3311, Demeditec Diagnostics GmbH; Kiel, Germany) and read by a FLUOstar Galaxy spectrometer (BMG LABTECH GmbH; Offenburg, Germany). As specified by the manufacturer, lower detection limits were 0.12 pg/mL (IL-1β), 0.13 pg/mL (IL-6), 0.12 pg/mL (IL-8), and 0.2 ng/mL (NE).

### Statistical Analyses

**Figure 2:**
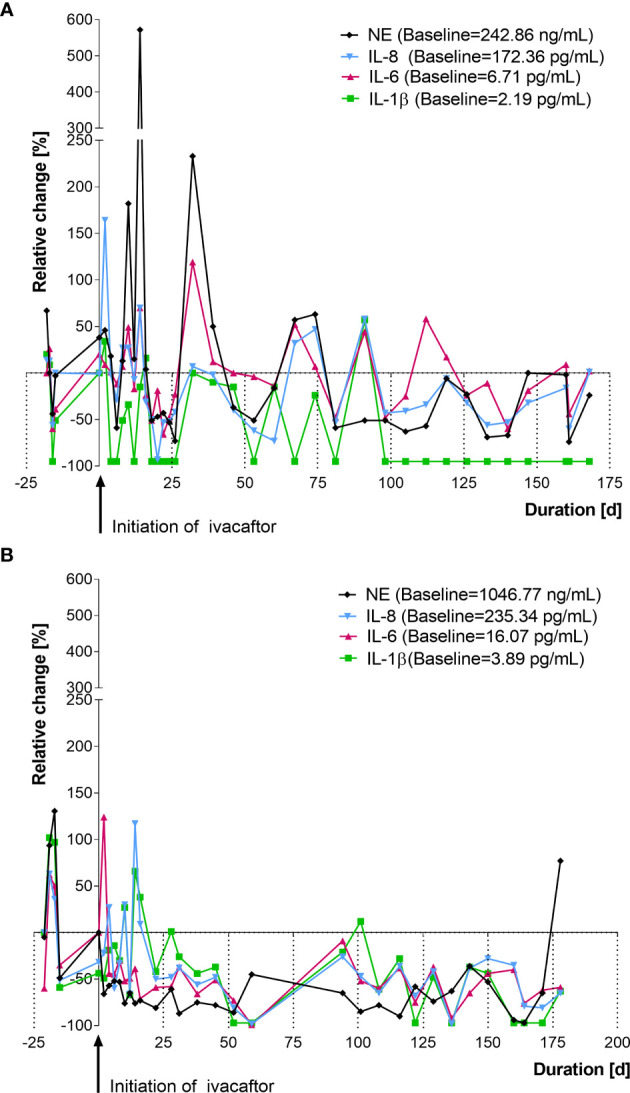
Changes in cytokines in nasal lavage after initiation of ivacaftor therapy (day 0) in two representative CF patients with a G551D mutation. **(A)** female, age: 27 years, follow up: 5.7 months. **(B)** male, age: 32 years, follow up: 5.8 months, values shown are relative changes to median of 5 [both **(A, B)**] measurements prior to ivacaftor. NE, neutrophil elastase; IL, interleukin.

Descriptive statistics were used to describe clinical characteristics of the study population. Linear mixed-effects models were performed to analyze overall trends of inflammatory markers. To smooth effects caused by outliers in inflammatory marker values, individual changes regarding cumulative periods for each patient were determined by calculating medians for the time periods before and after treatment initiation (i.e. regarding periods of 4, 8 and 12 weeks during treatment as shown in **Figure 2**). The resulting individual patients’ medians were used to calculate medians and interquartile ranges (IQR) of the entire cohort at the end of the above-mentioned time periods. Wilcoxon signed-rank test (two-tailed) was applied to determine changes in the aforementioned inflammatory mediators, sweat test, FEV_1_ and their statistical significance (using p<0.05) during the new therapy. All statistical analyses were performed with IBM SPSS 19 (SPSS Inc., Chicago, IL, USA); figures were created with GraphPad Prism (GraphPad Software Inc., La Jolla, CA, USA).

## Results

### Cohort Description

Ten participants (6 Jena/4 Innsbruck) were included, of whom 6 were female and 4 were male. At the start of the study, patients were aged 7-45 years (mean age: 16.55 ± 13.42 years). Results of sweat tests and lung function FEV_1_ were available only from 8 patients. Lung function at baseline, as obtained from those patients, ranged from 0.96 to 4.07 L (mean FEV_1_: 99.7 ± 20.3% predicted, median: 103.6% predicted, IQR=20.4% predicted).

### Analyses of Inflammatory Markers’ Time Trends

Mediator levels for each patient showed a rather dynamic behavior during the entire study period. **Figure 2** shows relative changes in IL-1β, IL-6, IL-8 and NE of two individual CF-patient receiving ivacaftor therapy. Given the highly variable behavior of the inflammatory mediators, baseline values for each patient were calculated as the median of the inflammatory marker levels before ivacaftor initiation (mean: 5.5 ± 3.9 samplings/patient).

**Figure 3:**
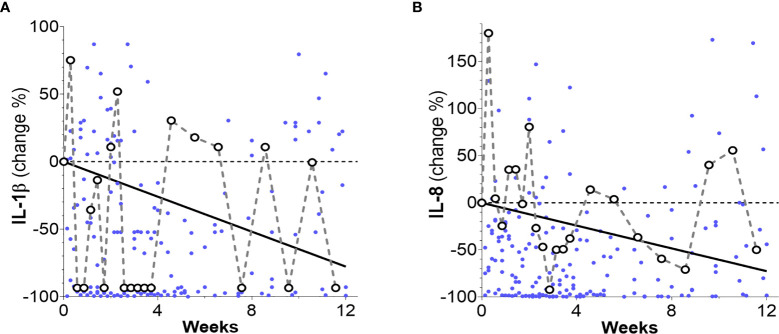
Percentage changes of patients’ inflammation markers levels (dots) and their overall time trends as obtained from linear mixed-effects models. **(A)** An overall decreasing trend for IL-1β is observed (solid line) over the whole 12-week time period (slope: -0.19 ± 0.09 pg/mL week, p = 0.042 and intercept 2.95 ± 0.49 pg/mL, p < 0.001). **(B)** An overall decreasing trend (solid line) was observed for IL-8 (slope: -11.48 ± 5.86 pg/mL week, p = 0.049 and intercept 189.76 ± 32.20 pg/mL, p < 0.001). Notice that for clarity, 16 data points surpassing the 100% level in **(A)** and 5 data points surpassing the 200% level in **(B)** are not shown.

Analyses of time trends of inflammation markers during the 12 weeks after therapy initiation were performed by using linear mixed-effects models. Downward overall trends with respect to baselines were found in the levels of inflammatory markers IL-1β (p = 0.042) and IL-8 (p = 0.045) (**Figures 3A, B** **)**. Cytokine IL-6 and Neutrophile Elastase levels showed no declining trend over the whole 12-week period.

### Change of Inflammatory Mediator Concentrations in Nasal Epithelial Lining Fluid

**Figure 4:**
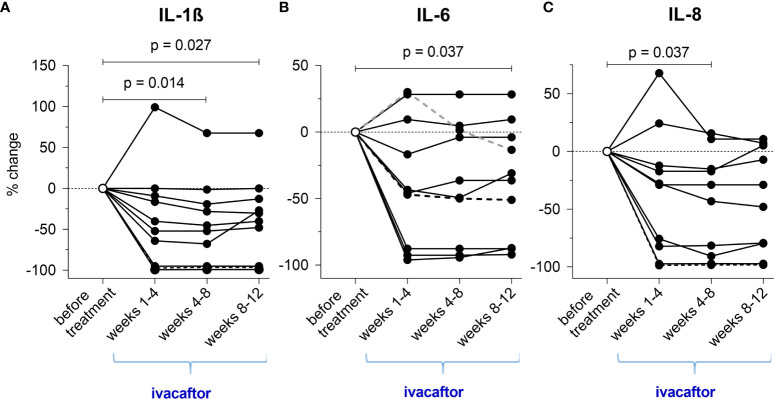
**(A–C)** Cumulative changes of inflammatory cytokines during the novel ivacaftor-therapy (regarding each periods of 4 weeks during treatment).

Comparison of medians before and during treatment revealed that ivacaftor significantly reduced inflammation in NL-fluid. From initiation of the new therapy to weeks 8-12, IL-1β declined 2.29-fold (from 2.97 [IQR=8.02] pg/mL to 1.30 [IQR=1.88] pg/mL, p=0.027) (**Figure 4A**) and IL-6 declined 1.13-fold (from 6.48 [IQR=7.97] pg/mL to 5.72 [IQR=5.86] pg/mL, p=0.037) (**Figure 4B**). IL-8 declined 3.53-fold (from 171.29 [IQR=481.49] pg/mL to 48.56 [IQR=122.94] pg/mL, p=0.037) until weeks 4-8. Although reduction of IL-8 continued after week 8 (57.15 [IQR=108.10] pg/mL), this change did not attain statistical significance in weeks 8-12 (**Figure 4C**). Similarly, changes in NE did not reach significance (from 346.22 [IQR=552.29] ng/mL to 285.47 [IQR=552.17] ng/mL).

### Comparison to Changes in Sweat Test and FEV

**Figure 5:**
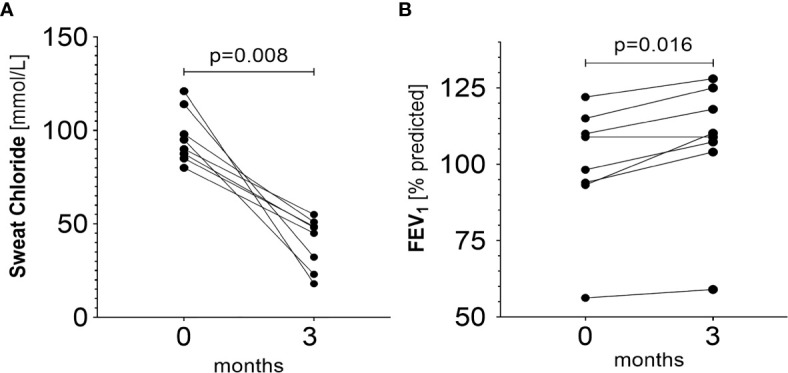
**(A)** Changes in sweat chloride prior to and during ivacaftor therapy (p = 0.008). Baseline mean: 96.31 ± 14.34 (median: 92.50, IQR=24.38) mmol/L. Mean after three months of therapy initiation: 40.10 ± 13.85 (median: 46.50, IQR=25.10) mmol/L. **(B)** Changes in FEV_1_ prior and during ivacaftor therapy (p=0.016). Baseline mean: 99.7 ± 20.3% predicted, (median: 103.6% predicted, IQR=20.4% predicted). Mean after three months of therapy initiation: 107.6 ± 21.4%pred (median: 109.6% predicted, IQR=18.4% predicted).

Sweat test and FEV_1_ support these results (**Figures 5A, B** **)**. Following the initiation of ivacaftor, mean chloride concentration in sweat declined from 96.31 ± 14.34 mmol/L to 40.1 ± 13.85 mmol/L (p=0.008) (iontophoresis with pilocarpine + chloridometry) and mean FEV_1_ improved from 99.7 ± 20.3% predicted to 107.6 ± 21.4% predicted (p=0.016).

## Discussion

### Key Findings

Our novel method for serial home-based sampling of NLF was applied to 10 CF patients carrying a G551D mutation and shows significant declines of IL-1β, 6 and 8 (2.27, 1.13 and 3.53-fold, respectively, p<0.05) during the first 3 months of treatment with ivacaftor. In agreement with these results, FEV_1_ increased from 99.7 ± 20.3% predicted to 107.6 ± 21.4% predicted, and mean chloride concentration in sweat declined from 96.31 ± 14.34 mmol/L to 40.1 ± 13.85 mmol/L.

### Potential of Serial NL Allowing Close Monitoring of Inflammatory Dynamics

This is the first study revealing direct effects on inflammatory markers in the NLF of CF patients with a G551D mutation following ivacaftor treatment. Our results from serial nasal lavages accord well to the longitudinal study by Hisert et al. (10), assessing inflammatory mediators in sputum from 12 adult CF patients carrying a G551D mutation: the authors found a significant decrease of IL-1β, IL-8 and NE levels in sputum during therapy with ivacaftor in a comparable cohort (n=12). Interestingly, decrease of inflammatory measures in sputum began during the first week of treatment, and continued to decline over 2 years, despite an initial mean FEV_1_% predicted of 64.2% and chronic infection of 8/12 patients with *P. aeruginosa* and of 2/12 with *B. cepacia*. Similarly, in the recent study by Jarosz-Griffiths et al., a steady decrease of IL-1β levels was observed in serum from a cohort (n=8) of F508del-homozygous CF patients during three months of the combination ivacaftor/tezacaftor therapy. In the same study, the combinations ivacaftor/tezacaftor and ivacaftor/lumacaftor in two CF-patient cohorts (n=8 and n=13, respectively) were reported to decrease levels of serum IL-18 (12). Furthermore, *in vitro*, the authors administered both approved dual combinations, i.e. ivacaftor with another CFTR potentiator, to LPS-ATP-stimulated monocytes from CF patients. They found that modulators down-regulate CF-related exaggerated pro-inflammatory responses differently: whereas IL-18 liberation significantly declines with each combination, only ivacaftor/tezacaftor reduces the IL-1β response.

CF, as a multi-organ disease, does not only lead to enhanced inflammation in the upper and lower airway system, as addressed. It also comprises gastrointestinal inflammation, e.g. reflected in elevated calprotectin in stool of people with CF. According to similar results, i.e. the reduction of inflammation in the upper airway in our study and the lower airways and lungs described by Hisert and Jarosz-Griffiths, ivacaftor was found to reduce faecal calprotectin in CF patients carrying a gating mutation (25).

In contrast to our findings, the authors of the “GOAL study” from 2014 did not detect significant changes of inflammatory markers in induced sputum (IS) from 14 patients with a G551D-mutation after six months of ivacaftor therapy (11). In a recent publication by the same research group, those results were expanded upon to compare changes in inflammatory markers from spontaneously expectorated sputum (ES) from an additional cohort (n=17) receiving ivacaftor therapy. Nevertheless, the authors did not find a reduction of inflammatory mediators in sputum obtained from these 31 patients and they state to have confirmed their findings that CFTR modulators lack anti-inflammatory effects (26). However, we estimate methodological limitations to cause these contradicting results. Firstly, in each patient, as little as one sample was taken before and another one 6 months after initiation of ivacaftor. This adds up to a total of 62 samples from the 31 patients included, i.e. 2 × 14 IS samples (initial study) + 2 × 17 ES samples (follow-up study). In contrast, we analyzed a mean of 25.9 ± 4.1 NL-fluid samples from each patient before and during a new therapy with ivacaftor (total: 259 NL samples) to allow a closer monitoring, an early detection of inflammatory changes and to smooth out possible measurement errors. Secondly, single assessment of cytokines in respiratory samples are known to be subject of high variances, e.g. influenced by multiple factors like circadian dynamics, effects of exercise, stress, and infection (27, 28). Variances in patients, which we attribute to such factors, are illustrated in **Figures 2A, B** including more than 50 samples taken from two patients from our cohort over a period of 200 days. They suggest that not only are high variances observed between patients but also within each patient from day to day, calling for an analysis of longitudinal nature. We would expect that similar variances would arise in sputum measurements, but no serial assessments of induced CF sputa have been performed yet, to our knowledge. Another possible factor causing the discrepancy with our results may be that sampling and processing of induced sputum requires complex SOPs (11) and immediate processing on-site, which can be challenging even in centers routinely processing IS. By inclusion of patients in different recruiting sites, this may be a limiting factor. In contrast, NL-fluid, just like BAL-fluid, can be directly frozen (e.g. with addition of protease inhibitors, as done in our study) and transferred on dry ice to a central laboratory.

## Conclusion

We conclude that CFTR modulators have an effect on inflammation in different organ systems involved by CFTR deficiency. In the airway system, serial sampling of NL-fluid, a sensitive non-invasive method that lacks complexity, allows to assess these effects. In contrast to the other mentioned methods to examine inflammation, it thus allows quantification of inflammatory changes on a day-to-day basis, and the patients can implement it even at home. Our approach can therefore add an interesting tool for repeated non-invasive sampling of airway-fluid to quantify early effects of CFTR modulators on airway inflammation.

## Data Availability Statement

The raw data supporting the conclusions of this article will be made available by the authors, without undue reservation.

## Ethics Statement

The studies involving human participants were reviewed and approved by Prof. Dr. Med. Ulrich Brandl, Dr. phil. Ulrike Skorsetz Universitätsklinikum Jena Ethik-Kommission Bachstraße 18 07743 Jena Germany. Written informed consent to participate in this study was provided by the participants’ legal guardian/next of kin. Written informed consent was obtained from the individual(s) for the publication of any potentially identifiable images or data included in this article.

## Author Contributions

Conceptualization: JGM and JH. Project administration: JGM, JH, and HE. Recruitment: CA, HE, JGM, and KW. Data acquisition: KW, CA, TL, and HE. Analysis and interpretation of data: CA, CZ, U-CH, TL, JGM, and FD. Manuscript writing: CA, JGM, JH, CZ, and FD. All authors contributed to the article and approved the submitted version.

## Funding

This Investigator Initiated Trial was supported by Vertex Pharmaceuticals, Boston, MA (IIS-2013-103094). This funding source had no role in the design, execution, analysis and interpretation of this study.

## Conflict of Interest

JGM reports grants and JGM and HE report speaker/board honoraria from Vertex, outside the submitted work.

The remaining authors declare that the research was conducted in the absence of any commercial or financial relationships that could be construed as a potential conflict of interest.
